# Adrenergic stress constrains the development of anti-tumor immunity and abscopal responses following local radiation

**DOI:** 10.1038/s41467-020-15676-0

**Published:** 2020-04-14

**Authors:** Minhui Chen, Guanxi Qiao, Bonnie L. Hylander, Hemn Mohammadpour, Xiang-Yang Wang, John R. Subjeck, Anurag K. Singh, Elizabeth A. Repasky

**Affiliations:** 1 Department of Immunology, Roswell Park Comprehensive Cancer Center, Elm and Carlton Streets, Buffalo, NY 14263 USA; 2 Department of Genetics, Virginia Commonwealth University, Richmond, VI 23298 USA; 3 Department of Cell Stress Biology, Roswell Park Comprehensive Cancer Center, Elm and Carlton Streets, Buffalo, NY 14263 USA; 4 Department of Radiation Oncology, Roswell Park Comprehensive Cancer Center, Elm and Carlton Streets, Buffalo, NY 14263 USA

**Keywords:** Oncology, Cancer, Cancer therapy, Radiotherapy

## Abstract

The abscopal effect following ionizing radiation therapy (RT) is considered to be a rare event. This effect does occur more frequently when combined with other therapies, including immunotherapy. Here we demonstrate that the frequency of abscopal events following RT alone is highly dependent upon the degree of adrenergic stress in the tumor-bearing host. Using a combination of physiologic, pharmacologic and genetic strategies, we observe improvements in the control of both irradiated and non-irradiated distant tumors, including metastatic tumors, when adrenergic stress or signaling through β-adrenergic receptor is reduced. Further, we observe cellular and molecular evidence of improved, antigen-specific, anti-tumor immune responses which also depend upon T cell egress from draining lymph nodes. These data suggest that blockade of β2 adrenergic stress signaling could be a useful, safe, and feasible strategy to improve efficacy in cancer patients undergoing radiation therapy.

## Abstract (web summary)

Abscopal responses in patients and preclinical models following radiation therapy are rare. Here the authors show that, in murine tumor models, reducing adrenergic stress or β2-adrenergic receptor signalling not only improves control of the irradiated tumor but significantly increases abscopal events.

## Introduction

Radiotherapy is one of the most common treatments for cancer. While its primary mechanism of action is thought to be direct damage to tumor cells in the field of irradiation^1^, it has been reported occasionally that some patients treated with local ionizing radiation therapy (RT), experience a regression of distant, non-irradiated tumor(s)^2^. This intriguing phenomenon, referred to as the “abscopal effect”^3,4^, is now thought to be due to the ability of RT to stimulate a systemic anti-tumor immune responses^5–8^. Indeed, as early as the 1970s, investigators conducting research on the efficacy of RT in mouse tumor models observed reduced efficacy in immunodeficient nude mice compared to immunocompetent mice^9^ pointing to a role for the adaptive immune response in the efficacy of RT. However, even in immunocompetent mice, it is clear that the abscopal response is disappointingly rare and unpredictable unless radiation is combined with immunotherapy^10–18^ or other therapies^19,20^. A more complete understanding of the interaction of RT and the immune response in pre-clinical models could reveal new strategies to increase the frequency of abscopal effects in the clinic, resulting in improved patient outcomes^21^.

Relative to this problem, during pre-clinical research using murine models of cancer and graft versus host disease, our lab has clearly demonstrated that Institutional Animal Care and Use Committee (IACUC)—mandated mild cool housing temperature for laboratory mice (typically ~22–23 °C) is sufficient to cause chronic adrenergic stress^22^ with activation of sympathetic nerves resulting in norepinephrine release needed for adaptive thermogenesis^23–25^. That this has a direct impact on the outcomes of studies involving a variety of disease models, including studies involving immune responses has been shown by comparing outcomes in mice housed under standard vs. thermoneutral temperatures^24–27^. Furthermore, our lab discovered that this chronic stress suppresses baseline anti-tumor immunity and accelerates tumor growth and metastasis^28,29^, while it also suppresses graft versus host disease following allogeneic hematopoietic stem cell transplantation^30,31^. When tumor-bearing mice are housed at their thermoneutral temperature (~30 °C), chronic adrenergic stress and norepinephrine production are significantly lowered, CD8^+^ T cell-dependent responses are significantly improved, while numbers and activities of immunosuppressive cells are reduced^29,32,33^. Alternatively, when β2- adrenergic receptor (AR) knockout (KO) mice were used, or if wildtype (WT) mice housed at 22 °C were given the pan-β-AR blocker propranolol, we observed a similar improvement in tumor growth control and increased CD8^+^ T cell infiltration was seen in mice housed at 30 °C^22,29,34^, suggesting that cool housing-induced adrenergic stress exerts its negative effects on the anti-tumor immune system through activation of β-ARs on immune cells.

We, and others, have also shown that adrenergic signaling directly increases tumor cell resistance to killing by chemotherapy and targeted therapies^22,35,36^, and our lab recently showed that standard housing temperature-induced adrenergic stress signaling increases intrinsic tumor cell resistance and constrains the anti-tumor efficacy of ionizing radiation^37^. However, neither we nor others have ever explored the relationship between the frequency of abscopal effects and housing temperature, or the role of host adrenergic stress signaling in general on anti-tumor immunity following RT. Here we use three different tumor models as well as physiological, pharmacological, and genetic strategies that reduce or block β2-adrenergic signaling to examine the impact of adrenergic stress signaling on the frequency of the radiation-induced abscopal effect. Our data reveal a surprising dependency of the overall efficacy of RT (against both irradiated and non-irradiated, distant tumors) on baseline chronic adrenergic stress due to housing temperature, and more specifically, to β2-AR signaling. These data provide a compelling rationale for testing RT in combination with β-blockers and/or other stress reducing strategies in cancer patients undergoing RT.

## Results

### Adrenergic stress blunts efficacy of RT and abscopal effects

**Fig. 1:**
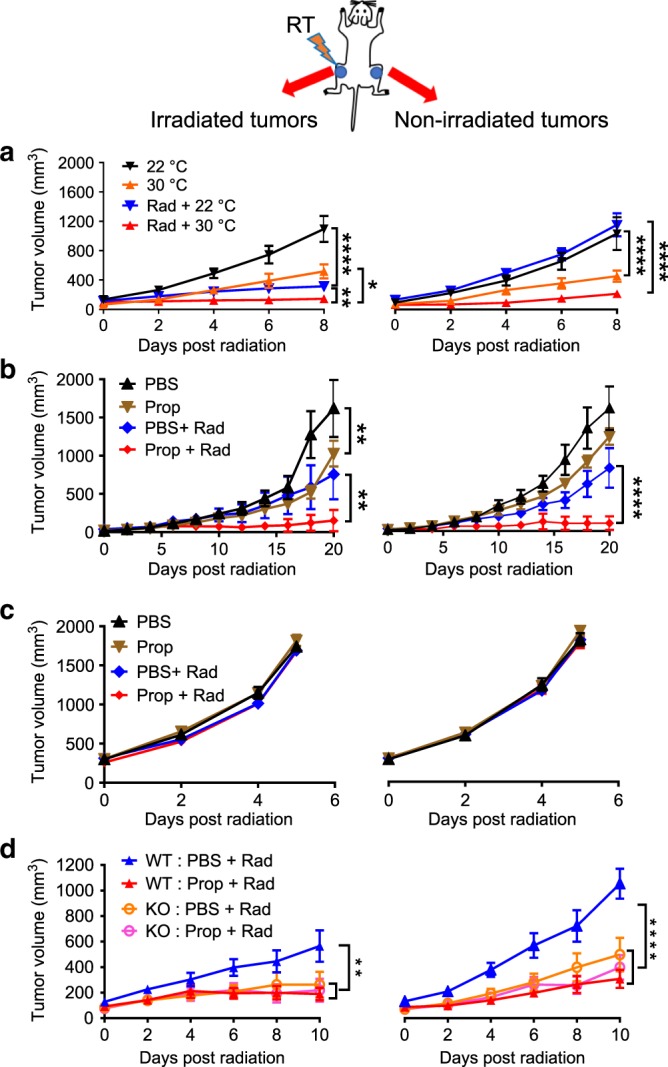
Reduction in β2-adrenergic signaling enhances the abscopal effect in a CT26 colon carcinoma model. BALB/c mice were implanted with CT26 tumors bilaterally; one tumor was irradiated. Tumor growth is shown for irradiated tumors (left) and non-irradiated tumors (right). **a** In mice housed at 22 °C (ST) or 30 °C (TT); **b** and **c** Comparison of tumor growth in BALB/c **b** or SCID mice **c**, which received no treatment, propranolol alone, radiation alone, or the combination; **d** Comparison of tumor growth in WT or β2-AR KO mice which received no treatment, propranolol alone, radiation alone, or the combination. Data are presented as mean ± SEM. *P < 0.05; **P < 0.01; ****P < 0.0001 (two-way ANOVA analysis). For **a**, *n* = 9 biologically independent mice in 22 °C, 30 °C and Rad + 30 °C groups, *n* = 7 biologically independent mice in Rad + 22 °C group; for **b**, *n* = 6 biologically independent mice in PBS, Prop, and PBS + Rad groups, *n* = 5 biologically independent mice in Prop + Rad group; for **c**, *n* = 10 biologically independent mice in all groups; for **d**, *n* = 6 biologically independent mice in WT PBS + Rad and KO PBS + Rad groups, *n* = 7 biologically independent mice in WT Prop + Rad group, *n* = 8 biologically independent mice in KO Prop + Rad group.

We previously reported that the standard cool housing temperature mandated for laboratory mice accelerates tumor growth and metastasis and suppresses anti-tumor immunity by subjecting mice to chronic adrenergic stress^28,29^. Here, we investigated the effect of adrenergic stress on the efficacy of local RT in three different tumor types (CT26.CL25 colon tumors in BALB/cAnNcr (BALB/c) mice and B16 melanoma in C57BL/6NCr (C57BL/6) mice, and 4T1 mammary tumors in BALB/c mice—a clinically relevant metastatic model). We also used three different strategies for manipulating adrenergic stress: physiological, pharmacological, and genetic. We began by testing whether the efficacy of ionizing radiation and, specifically, the abscopal effect^5,8,38^, is influenced by the physiological stress induced by housing mice at the standard (IACUC-mandated) housing temperature. As shown in Fig. 1a, we maintained mice at either 22 °C (the standard housing temperature, ST) used at Roswell Park or acclimated them to 30 °C (the thermoneutral temperature, TT, for laboratory mice which alleviates cold stress and results in reduced levels of norepinephrine (NE)) for at least 3 weeks. We then implanted either CT26.CL25 colon tumor cells (hereafter referred to as CT26) or B16 melanoma cells in both hind limbs. When the tumors became palpable, one tumor in each mouse was exposed to local RT (6 Gy for CT26, 20 Gy for B16). We selected these radiation doses for this study as they produce a reproducible slowing of tumor growth but are suboptimal for each tumor type and do not result in complete tumor control nor in abscopal effects. As we have shown previously, tumors in mice housed at ST grew significantly faster than those housed at TT (which reduces adrenergic stress). Indeed, the tumor growth inhibition seen by simply housing mice at TT was similar to that achieved by treatment of mice at ST with radiation; the effect of radiation was further enhanced when mice were housed at TT (CT26, Fig. 1a left; B16, Supplementary Fig. 1a left). By comparison, in mice housed at ST, RT had no effect on the contralateral, non-irradiated tumor, while there was a reduced growth rate in those tumors in all of the mice housed at TT mice, i.e., an abscopal effect was generated (CT26, Fig. 1a right and Supplementary Fig. 1c left; B16, Supplementary Fig. 1a right and b). That this abscopal effect is mediated by immune activity is supported by the presence of an increased number of intratumoral CD8^+^ T cells, especially CD8^+^ effector/memory T cells (Supplementary Fig. 1c middle and right) in the contralateral, non-irradiated tumors of mice housed at TT.

Since we know that housing at 22 °C increases adrenergic stress, we next used a pharmacological strategy, treatment of mice with the β-AR antagonist propranolol (Prop), to test the role of β-ARs in response to radiation. This experiment had four groups of mice at 22 °C: (1) phosphate-buffered saline (PBS) alone, (2) Prop alone, (3) PBS + RT, and (4) Prop + RT. We found that compared to PBS and RT alone, Prop plus RT slowed tumor growth of both the irradiated and contralateral, non-irradiated tumors (Fig. 1b), indicating that a blockade of adrenergic signaling greatly improves efficacy of RT in mice housed at ST. We repeated this experiment with SCID (C.B. Igh-1b Icr Tac Prkdc scid) mice, which are characterized by an absence of functional T and B cells, and neither radiation nor Prop had any effect on tumor growth of the irradiated tumor or the contralateral, non-irradiated tumor (Fig. 1c), indicating that the overall effect of this dose of radiation on both the primary and contralateral tumor depends upon an intact immune system.

To further dissect the role of β-adrenergic signaling in the efficacy of RT, we utilized a genetic strategy with β2-AR KO mice and found that the growth of CT26 irradiated and contralateral non-irradiated tumors β2-AR KO mice with or without Prop, was similar to that seen in irradiated WT mice given Prop (Fig. 1d), suggesting that the improved efficacy and abscopal effect is dependent on β2-ARs. Taken together, these data support the idea that adrenergic signaling is a critical pathway by which the efficacy of RT against both irradiated and non-irradiated distant tumors is regulated.

### Impact of reducing β-AR signaling depends on CD8^+^ T cells

**Fig. 2:**
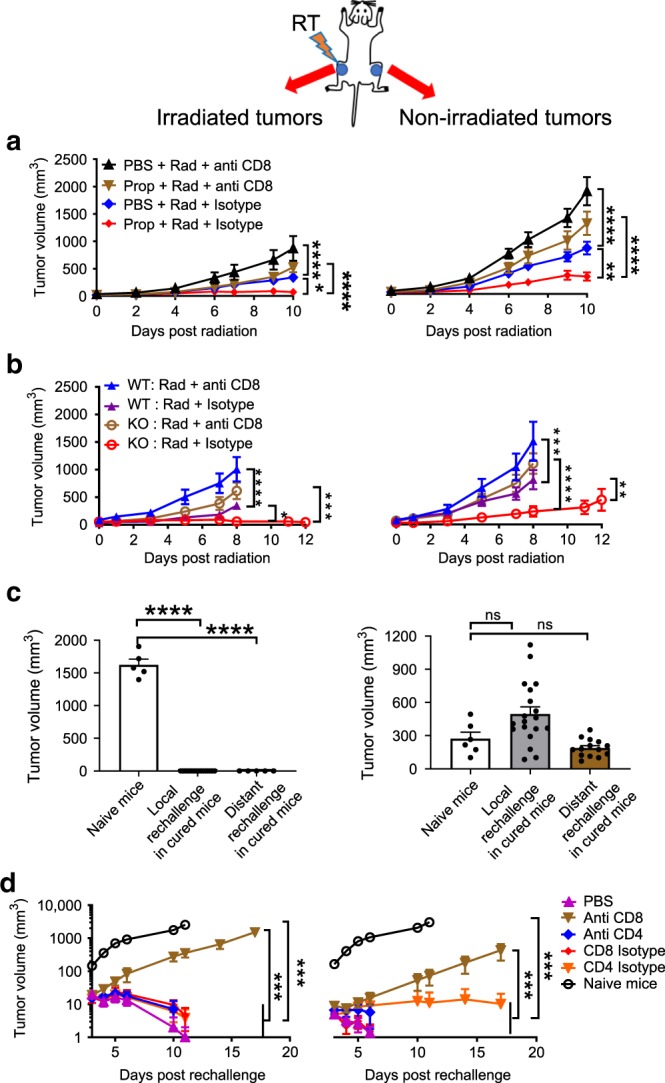
CD8^+^ T cells are crucial for the improved abscopal effect achieved by blocking/absence of β2-adrenergic signaling in CT26 colon carcinoma model. **a** and **b** Growth of irradiated (left) and non-irradiated (right) tumors in irradiated WT mice treated with or without propranolol **a** and irradiated WT mice and β2-AR KO mice **b**, which were depleted of CD8^+^ T cells. **c** The tumor growth of local and distant rechallenges with CT26 (left) and 4T1 (right) in mice cured by radiation plus propranolol compared with naïve mice. **d** The tumor growth (a log scale) of local (left) and distant (right) rechallenges with CT26 in mice cured by radiation and propranolol depleted of CD8^+^ or CD4^+^ T cells. Data are presented as mean ± SEM. *P < 0.05; **P < 0.01; ***P < 0.001; ****P < 0.0001; ns = not significant (two-way ANOVA analysis for **a**, **b**, **d**; one-way ANOVA analysis for **c**). For **a**, *n* = 10 biologically independent mice in all groups; for **b**, *n* = 9 biologically independent mice in WT Rad + anti-CD8 and WT Rad + isotype groups, *n* = 8 biologically independent mice in KO Rad + anti-CD8 group, *n* = 10 biologically independent mice in KO Rad + isotype group; for CT26 model in **c**, *n* = 5 biologically independent mice in naïve mice group, *n* = 16 biologically independent mice in local rechallenge in cured mice group, *n* = 5 biologically independent mice in distant rechallenge in cured mice group; for 4T1 model in **c**, *n* = 6 biologically independent mice in naïve mice group, *n* = 19 biologically independent mice in local rechallenge in cured mice group, *n* = 14 biologically independent mice in distant rechallenge in cured mice group; for **d**, *n* = 6 biologically independent mice in PBS, anti-CD8, anti-CD4, CD8 isotype, and CD4 isotype groups, *n* = 5 biologically independent mice in naïve mice group.

Data obtained from SCID mice (Fig. 1c) strongly suggest that the anti-tumor immune response is a major target of reducing or blocking adrenergic signaling in combination with RT. Supporting this assumption, depletion of CD8^+^ T cells in BALB/c mice treated with radiation and Prop greatly reduced the improved efficacy seen against both irradiated and distant non-irradiated tumors (Fig. 2a). Moreover, CD8^+^ T cell depletion also eliminated the improved tumor control seen in irradiated β2-AR KO mice (Fig. 2b). These data suggest that CD8^+^ T cells are critical for the improved abscopal effect seen by blocking/absence of β2-adrenergic signaling. This critical role for CD8^+^ T cells also suggested that the effects we observed are tumor antigen-specific. To test this assumption, BALB/c mice cured by Prop and radiation (with both irradiated and distant tumors having disappeared for at least 2 months) were rechallenged with either the same tumor used earlier in these mice, CT26 or a different tumor, 4T1. Figure 2c showed that compared to naïve mice, the tumor growth of local and distant CT26 tumor rechallenge was inhibited in all previously cured mice (Fig. 2c, left). When mice previously cured of CT26 tumor were subsequently rechallenged with 4T1 cells, growth of 4T1 tumor was not inhibited (Fig. 2c, right), indicating that the growth inhibition was tumor-specific.

To investigate which immune cells are primarily responsible for the control of subsequent rechallenges, cured mice were treated with CD8^+^ or CD4^+^ depleting antibody prior to re-injection with CT26 tumor cells. We found that the cured mice depleted of CD8^+^ T cells, but not CD4^+^ T cells, did not reject either local or distant rechallenges (Fig. 2d), indicating that CD8^+^ T cells are the key players for induction of memory responses against local and distant tumor rechallenges. As a result of these experiments, we determined that CD8^+^ T cells are crucial for the improved abscopal effect by blocking/absence of β2-adrenergic signaling and also for inducing memory response in a tumor-specific manner.

### Immune status following RT is dependent upon β2-AR signaling

**Fig. 3:**
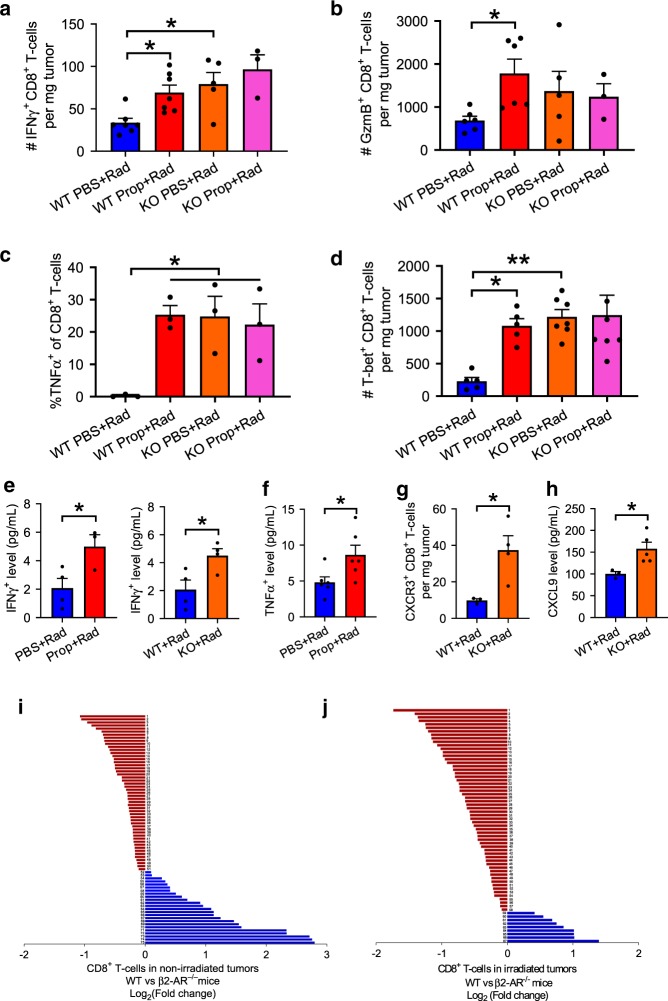
Blocking/absence of β2-adrenergic signaling increases expression of genes involved in enhancing the cell-mediated anti-tumor immune response and T cell egress in non-irradiated CT26 tumors. Immune analyses were performed in non-irradiated tumors of irradiated WT or β2-AR KO mice treated with or without propranolol. Reduction or absence of β2-adrenegic signaling increased the expression of important effector molecules in CD8^+^ T cells including: **a** IFNγ^+^, **b** GzmB^+^, **c** TNFα^+^, and **d** T-bet^+^. The level of IFN-γ was detected in serum of irradiated WT mice treated with or without propranolol (**e**, left panel) or irradiated WT and β2-AR KO mice (**e**, right panel) as well as TNFα in irradiated mice treated with or without propranolol **f**. The expression of CXCR3 in non-irradiated tumors **g** and the level of CXCL9 in serum **h** was detected in irradiated WT or β2-AR KO mice. **i** and **j** Gene profiles with NanoString in CD8^+^ T cells (data are presented as Log_2_ (fold change of WT/KO)) of the non-irradiated tumors **i** and irradiated tumors **j** from WT and β2-AR KO mice which had been given radiation to the contralateral tumors. Detailed information is shown in Supplementary data 1. Data are presented as mean ± SEM. *P < 0.05; **P < 0.01 (one-way ANOVA analysis for **a**–**d**; Student’s *t* test **a**nalysis for **e**–**h**). For **a**, *n* = 7 biologically independent mice in WT PBS + Rad and WT Prop + Rad groups, *n* = 5 in KO PBS + Rad group, *n* = 3 in KO Prop + Rad group; for **b**, *n* = 6 biologically independent mice in WT PBS + Rad and WT Prop + Rad groups, *n* = 5 in KO PBS + Rad group, *n* = 3 in KO Prop + Rad group; for **c**, *n* = 3 biologically independent mice in all groups; for **d**, *n* = 5 biologically independent mice in WT PBS + Rad and WT Prop + Rad groups, *n* = 7 in KO PBS + Rad and KO Prop + Rad groups; for **e**, *n* = 4 biologically ind**e**pendent mice in PBS + Rad, WT + Rad, and KO + Rad groups, *n* = 3 in Prop + Rad group; for **f**, *n* = 6 biologically independent mice in all groups; for **g**, *n* = 3 biologically independent mice in WT + Rad group, *n* = 4 in KO + Rad group; for **h**, *n* = 3 biologically independent mice in WT + Rad group, *n* = 5 in KO + Rad group; for **i** and **j**, *n* = 8 biologically independent mice in WT + Rad and KO + Rad groups.

To explore the underlying cellular events by which reduced β2-adrenergic signaling enhances anti-tumor immunity, we enumerated effector CD8^+^ T cells in tissues isolated from mice in the experiments presented above with flow cytometry. In the distant non-irradiated tumors from irradiated WT mice treated with Prop as well as irradiated β2-AR KO mice treated with or without Prop, interferon gamma^+^ (IFNγ^+^), granzyme B^+^ (GzmB^+^), tumor necrosis factor^+^ (TNFα^+^), and T-box transcription factor^+^ (T-bet^+^) intra-tumoral effector CD8^+^ T cells significantly increased (Fig. 3a–d, Supplementary Fig. 2a), while M2 macrophages decreased (Supplementary Fig. 3). This suggests that blocking of β2-adrenergic signaling enhances T cell-mediated anti-tumor immune responses in distant non-irradiated tumors while reducing the presence of M2 macrophages which are been shown to be immunosuppressive and pro-tumorigenic^39–41^. To assess the production of cytokines, which is an indicator of overall immune status, we measured the levels of IFNγ and TNFα in the serum from the mice treated with β-blocker and radiation as well as the β2-AR KO mice treated with radiation. Figure 3e and f showed that both IFNγ and TNFα levels increased in these mice. We also checked chemokine receptor C-X-C chemokine receptor type 3 (CXCR3) on CD8^+^ T cells in distant non-irradiated tumor and its ligand C-X-C motif chemokine 9 (CXCL9) in serum, which are associated with CD8^+^ T cell egress^42–44^, and found that both CXCR3 and CXCL9 increased in irradiated β2-AR KO mice compared to irradiated WT mice (Fig. 3g and h), supporting the possibility that absence of β2-AR signaling enhances the migration of CD8^+^ T cells by CXCR3/CXCL9 interactions.

To further explore the impact of adrenergic signaling on these and other molecules, we compared gene expression in CT26 tumors implanted bi-laterally in both WT mice and β2-AR KO mice, followed by radiation to just one of the tumors in each mouse. Shown in Fig. 3i and j is the Log_2_ (fold change of the ratio of WT/KO) from CD8^+^ T cells. For the non-irradiated tumor in β2-AR KO mice (Fig. 3i), we noted a significant increase in expression of several genes including *Cd28*, *Il2*, and *Zap70*; genes showing the greatest decrease included genes which can inhibit effector function (including *Il6* and *Il10*). For a complete list of differentially expressed genes, see Supplementary data 1. Importantly, IFNγ-related genes *Cxcr3* increased in the non-irradiated tumors of β2-AR KO mice, confirming the serum expression data above and supporting the idea that T cell migration from draining lymph node (LN) to tumors is enhanced in the absence or reduction of adrenergic signaling.

For irradiated tumors of β2-AR KO mice, expression of genes essential for effector function, including *Cd28*, *Cd86*, *Il2*, *Gzmb*, *Cd3d*, and T cell migration, including *Sele*, *Csf2*, *Cxcr6*, *Cd44*, showed increased expression (Fig. 3j and Supplementary data 1). *Cxcr3* and its ligand *Cxcl9* were also seen to be increased in irradiated tumors of β2-AR KO mice. The significantly changed genes were used as input for Gene Ontology (GO) and pathway analysis. The upregulated genes in non-irradiated tumor of β2-AR KO mice in the red module in Supplementary Fig. 4a were related to T cell receptor signaling pathway [false discovery rate (FDR) = 1.41 × 10^−4^], receptor signaling pathway via Janus kinase-signal transducer and activator of transcription (JAK-STAT) (FDR = 1.70 × 10^−6^), positive regulation of tyrosine phosphorylation of STAT protein (FDR = 1.62 × 10^−5^), positive regulation of nuclear factor kappa B (NFκB) transcription factor activity (FDR = 5.91 × 10^−4^), positive regulation of interleukin 2 (IL-2) biosynthetic process (FDR = 3.25 × 10^−6^), positive regulation of activated T cell proliferation (FDR = 3.76 × 10^−5^). On the other hand, the red module in Supplementary Fig. 4b also showed that the upregulated genes in irradiated tumor of β2-AR KO mice were related to similar important regulatory functions.

### Role of tumor draining LNs in enhanced abscopal effects

**Fig. 4:**
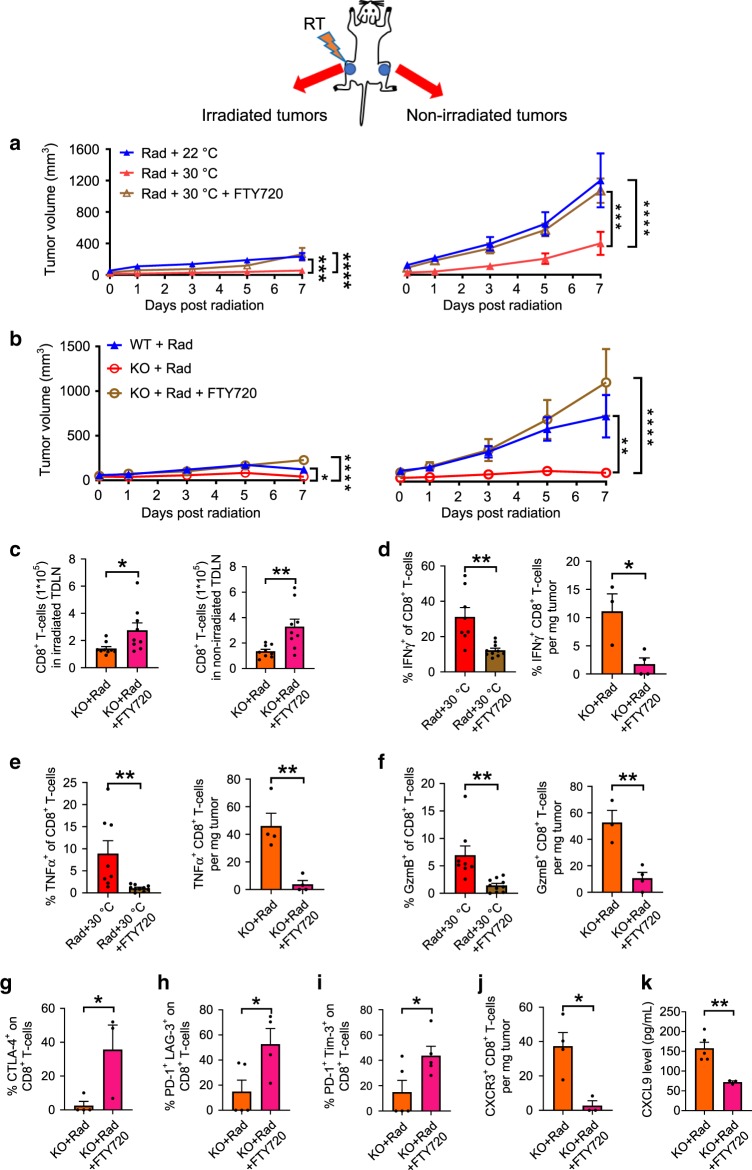
β2-adrenergic signaling modulates the abscopal effect via the egress of CD8^+^ T cells from the LN in the CT26 colon carcinoma model. Growth of irradiated tumors (left) and non-irradiated tumors (right) in the mice housed at 22 °C (ST) or 30 °C (TT) that did or did not receive FTY720 **a**; or in WT or β2-AR KO mice which were irradiated and then did or did not receive FTY720 **b**; **c** CD8^+^ T cells in the draining LN of irradiated (left) and non-irradiated (right) tumors in irradiated β2-AR KO mice treated with/without FTY720; **d**–**f** Data from irradiated WT mice housed at 30 °C (left panels) and β2-AR KO mice (right panels) which received radiation with/without FTY720: expression of IFNγ^+^ **d**, TNFα^+^ **e**, and GzmB^+^ **f**; **g**–**i** the expression of CTLA-4^+^ **g**, PD-1^+^ and LAG-3^+^ **h**, PD-1^+^, and Tim-3^+^ **i** in non-irradiated tumors from irradiated β2-AR KO mice treated with/without FTY720. The expression of CXCR3 in non-irradiated tumors **j** and the level of CXCL9 in serum **k** was detected in irradiated β2-AR KO mice treated with/without FTY720. Data are presented as mean ± SEM. *P < 0.05; **P < 0.01; ***P < 0.001; ****P < 0.0001 (two-way ANOVA analysis for **a**, **b**; Student’s *t* test analysis for **c**–**k**). For **a**, *n* = 10 biologically independent mice in all groups; For **b** and **c**, *n* = 9 biologically independent mice in all groups; For **d** and **f**, *n* = 8 biologically independent mice in Rad + 30 °C group, *n* = 10 biologically independent mice in Rad + 30 °C + FTY720 group, *n* = 3 biologically independent mice in KO + Rad group, *n* = 4 biologically independent mice in KO + Rad + FTY720 group; For **e**, *n* = 8 biologically independent mice in Rad + 30 °C group, *n* = 10 biologically independent mice in Rad + 30 °C + FTY720 group, *n* = 4 biologically independent mice in KO + Rad group, *n* = 4 biologically independent mice in KO + Rad + FTY720 group; For **g** and **j**, *n* = 4 biologically independent mice in KO + Rad group, *n* = 3 biologically independent mice in KO + Rad + FTY720 group; For **h**, *n* = 5 biologically independent mice in KO + Rad group, *n* = 4 biologically independent mice in KO + Rad + FTY720 group; For **i**, *n* = 5 biologically independent mice in two groups; For **k**, *n* = 5 biologically independent mice in KO + Rad group, *n* = 3 biologically independent mice in KO + Rad + FTY720 group.

A current proposed mechanism by which radiation can increase anti-tumor immunity suggests that damage to the tumor from ionizing radiation increases antigen availability and uptake by dendritic cells which then travel to draining LNs where they activate naïve, antigen-specific T cells. Effector T cells must then re-enter the circulation from the draining LNs to target antigen-expressing tumor cells^38,45^. However, recent studies show that β2-AR adrenergic signaling in activated T cells inhibits their exit from LNs and suggests that this occurs by enhancement of retention promoting signals^46,47^. Thus, to investigate the role of T cell egress from draining LNs in the mechanism underlying enhanced abscopal effects achieved by combining radiation with reduced β-AR signaling, we used both WT mice (at TT) or β2-AR KO mice (at ST) as models of reduced AR signaling. Mice with bilateral tumors received RT to one tumor and were treated with FTY720, a sphingosine 1-phosphate receptor (S1PR) agonist which is phosphorylated by cellular sphingosine kinases and binds to S1PR resulting in receptor internalization and degradation. This drug is well recognized to prevent T cell egress from lymphoid tissues leading to decreased numbers of lymphocytes in non-lymphoid peripheral tissues^48–50^. We observed that FTY720 treatment resulted in a significant loss of the enhanced tumor control seen by housing mice at 30 °C (CT26, Fig. 4a; B16, Supplementary Fig. 5a, b). Similarly, the enhanced immune control of tumors induced by RT in β2-AR KO mice was also lost in mice treated with FTY720 (Fig. 4b). Analysis of CD8^+^ T cells by flow cytometry confirmed increased numbers of CD8^+^ T cells retained in the draining LNs of both irradiated and distant non-irradiated tumors in KO mice treated with FTY720 (Fig. 4c). These results support a role for T cells which had been in the draining LN (and not simply present in the nearby tissues or blood) in generating improved systemic immunity following radiation in the absence of adrenergic stress signaling.

To further our understanding of how FTY720 and adrenergic signaling influences the immune landscape in tumors, we characterized expression of various effector molecules and co-inhibitory receptors on Day 7 post-RT using flow cytometry in mice treated or non-treated with FTY720. As shown in Fig. 4d–f (left panel), FTY720 treatment was associated with fewer IFNγ^+^, TNFα^+^, and GzmB^+^ CD8^+^ T cells in these tumors compared with those of control mice housed at 30 °C. Similar results were obtained from irradiated β2-AR KO mice (Fig. 4d–f right panel), however, we also found that expression of co-inhibitory molecules (cytotoxic T-lymphocyte-associated protein 4 (CTLA-4), programmed cell death-1 (PD-1), lymphocyte-activation gene 3 (LAG-3) and T cell immunoglobulin and mucin-domain containing-3 (Tim-3)) was significantly increased (Fig. 4g–i). Together, these data support the possibility that adrenergic signaling sequesters effector T cells in the draining LN^46,47^, and reversal of suppression allows migration of these T cells into the tumor thereby allowing development of an abscopal response. Moreover, as shown in Fig. 4j and k and Supplementary Fig. 2b, CXCR3^+^CD8^+^ T cells (in the non-irradiated tumor) and CXCL9 (in the serum) were decreased in FTY720 treated, irradiated β2-AR KO mice compared to controls, suggesting that CXCR3/CXCL9 may play an important role in CD8^+^ T cell migration to tumors in the absence of β2-AR expression. Similar results were also observed in B16 model (Supplementary Fig. 6a–d). Taken together, these data lead to the speculation that one mechanism by which β2-adrenergic signaling suppresses the abscopal effect is by retention of CD8^+^ T cells within the tumor draining LNs, as has been seen in the experimental autoimmune encephalomyelitis model of autoimmune disease^46^. However, this potential mechanism is based on correlative data so far, and further study is required to establish a causative role for adrenergic stress in specific patterns of immune cell migration or trafficking to the tumor microenvironment.

### β-blocker improves combination of RT and checkpoint blockade

After determining that β-blocker enhances abscopal effect and is associated with decreased PD-1 expression on CD8^+^ T cells, we then investigated whether β-blocker can further improve radiation responses, especially abscopal effect, in anti-PD-1 checkpoint blockade immunotherapy. The mice were implanted with CT26 tumor cells and treated with Prop or PBS as described before. After local radiation of one tumor, the mice were treated with six doses of anti-PD-1 or isotype control antibodies. A detailed experimental design is shown in Supplementary Fig. 7a. Comparison of tumor growth (Supplementary Fig. 7b) showed that compared to PBS, Prop significantly enhanced the tumor control of irradiated and non-irradiated tumors in the mice treated with anti-PD-1 antibody, suggesting that adrenergic stress interferes with the effects of the combination of radiotherapy and immunotherapy, which can be overcome by the treatment of β-blocker.

### β-blocker reduces metastatic spread after local RT

**Fig. 5:**
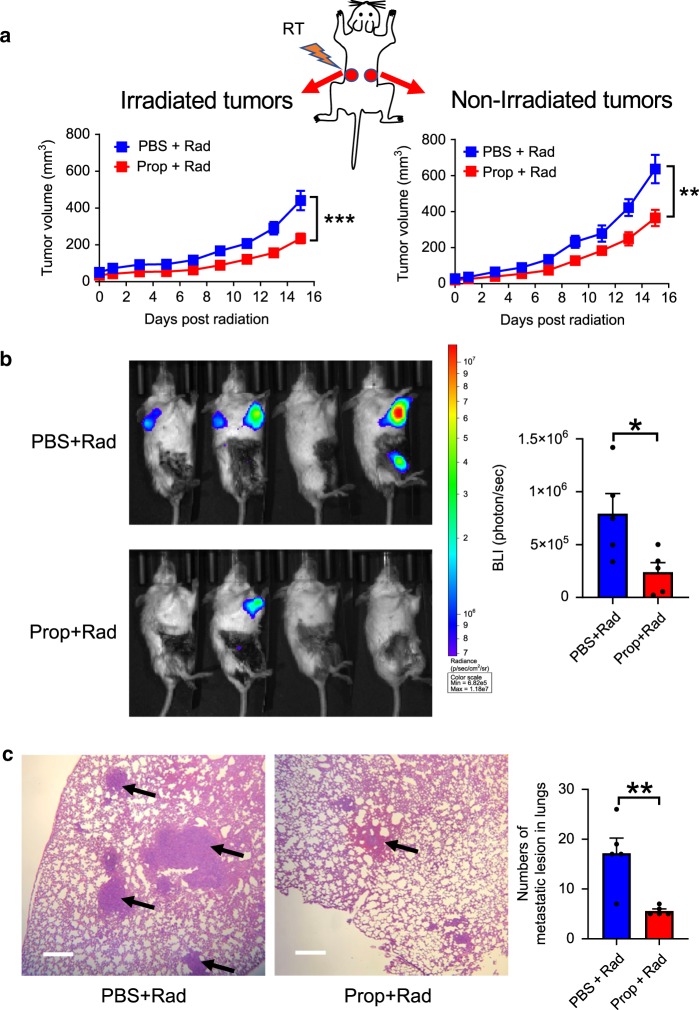
Blockade of β-adrenergic signaling reduces spontaneous metastases in the 4T1-luc breast carcinoma model. **a** Tumor growth of irradiated tumors (left) and non-irradiated tumors (right) is shown. Data are presented as mean ± SEM. **P < 0.01; ***P < 0.001 (two-way ANOVA analysis). *n* = 12 biologically independent mice in PBS + Rad group and *n* = 18 biologically independent mice in Prop + Rad group. On Day 33 post tumor implantation, bioluminescent images of luciferase activity **b** were acquired and histologic analysis of lung tissues was shown in **c**. Staining with eosin hematoxylin was performed (×40 magnification, scale bars = 500 µm) and black arrows showed metastatic lesions. Data are presented as mean ± SEM. *P < 0.05; **P < 0.01 (Student’s *t* test analysis). For **b** and **c**, *n* = 5 biologically independent mice in all groups.

Thus far, we have used implantable tumor models to investigate the impact of β-AR signaling on control by radiation of both irradiated and non-irradiated, distant tumors. Naturally occurring metastatic tumors offer an important opportunity to study factors which affect the abscopal effect. Thus, to investigate whether β-AR blockade affects metastases after radiation to the primary tumor, we employed the 4T1-Luc model to repeat the experiment with the treatment of Prop and radiation. 4T1 tumors exhibit metastatic spread to the lungs following their orthotopic implantation to the mammary glands of mice^32,51^. As shown in Fig. 5a, compared with PBS group, β-blocker Prop treatment significantly improved the tumor control of both irradiated and non-irradiated mammary tumors after 12 Gy local radiation to one of the orthotopically implanted tumors. On Day 33 post tumor implantation, bioluminescence imaging (BLI) of luciferase activity (Fig. 5b) showed that Prop combined with radiation given earlier to an orthotopically implanted mammary tumor also reduced lung metastases. Moreover, analysis of lung tissue morphology on haemotoxylin and eosin (H&E)-stained histologic sections also indicated fewer metastatic lesions in the Prop-treated group (Fig. 5c). These data suggest that blockade of β-AR signaling reduces spontaneous metastases after radiation to the primary tumor.

## Discussion

RT used as a single agent is now thought to promote immunity against tumors, including increased generation of antigen-specific effector T cells. However, a common belief is that radiation alone is not sufficient to induce a therapeutically effective, systemic T cell response capable of controlling distant, non-irradiated tumors (i.e., an abscopal effect). The data presented here provide a counter-argument to this widespread assumption.

Three conclusions can be drawn from the data presented here. First, the baseline adrenergic stress caused by cool housing temperature mandated for laboratory mice for decades is immunosuppressive and has led to a significant underestimation of the natural ability of radiation alone to elicit an abscopal effect. This point alone should alert the radiation biology community to the dependence of experimental outcomes on physiological and environmental factors which are largely ignored by researchers. Using a combination of pharmacological and genetic approaches, our second finding is that the mechanism by which cool housing temperature influences the abscopal effect is via AR signaling leading to immunosuppression. This includes a demonstration of enhanced control of metastatic tumors in the 4T1 mammary tumor model where radiation was delivered to the primary tumor in the presence of Prop. Third, the enhancement of the abscopal effect achieved by reducing adrenergic signaling is dependent upon CD8^+^ T cells, and perhaps the response of other immune cell types, and is associated with significant beneficial changes in the immune status of irradiated and non-irradiated tumors. This results in an immune system that is more capable of controlling tumors outside the field of radiation. Together, these data reveal an unexpected degree of control by the sympathetic nervous system over the efficacy of radiation, both in irradiated tumors and those at distant non-irradiated sites. Thus, adrenergic stress signaling in the tumor may be included with other known physiological factors that influence the efficacy of RT, such as intratumoral hypoxia or metabolic activity^52–54^. These data also provide additional evidence for the importance of tumor immunity in mediating the overall outcome of RT and abscopal events.

The precise mechanisms by which blockade or absence of β-AR signaling enhances systemic anti-tumor immunity following local radiation remain to be established. Data presented here show a critical role for CD8^+^ T cells with upregulation of T-bet transcription, increased secretion of IFNγ, TNFα and GzmB and upregulation of CXCR3/CXCL9 signaling, which is required for effector T cell migration across tumor vessels^55^. This adds to our previously published data (in non-radiation settings) that blockade of β-AR signaling increases metabolic activity during CD8^+^ T cell activation^33^. Further, we have observed that the addition of Prop reduces the frequency of immunosuppressive M2 macrophages in the non-irradiated tumor following radiation. Macrophages have previously been shown to be regulated by adrenergic stress^56,57^ and M2 macrophages in particular have been linked to enhanced immunosuppression^39–41^. This is consistent with our other data that the blockade of adrenergic stress suppresses the recruitment, survival, and function of myeloid-derived suppressor cells (MDSCs) and reduces the frequency of intratumoral regulatory T cells (Tregs)^29,32,58^. Thus, the combination of enhanced activity of CD8^+^ T cells combined with reduced immunosuppression in mice resulting from reduced β-AR signaling could result in a major tilt of the immune balance toward systemic tumor eradication following local radiation.

Preclinical models of many tumor types have shown that radiation can increase recognition of malignant cells through a variety of mechanisms including enhanced antigen uptake and presentation by dendritic cells^59,60^. Our data show that the enhanced abscopal effect following reduction or blockade of adrenergic signaling is dependent on T cell egress from tumor draining LNs. This supports a model^38,45^ in which radiation stimulates antigen-dependent activation of naïve T cells in draining LNs, which then must exit the LNs and migrate to the tumor microenvironment. However, these data are still indirect and correlative. Experiments which directly demonstrate a causal regulation of T cell, migration patterns and trafficking to the tumor microenvironment by adrenergic stress are still needed.

Other questions arise from these data. For example, what is the impact of adrenergic stress on the efficacy of different radiation schedules (e.g., fractionated vs. hypofractionated) and on anti-tumor immunity^61^? Here, we used single fraction doses that are known to be suboptimal in controlling tumor growth within the field of irradiation and do not result in abscopal effects. This is an important question to answer because in the clinic, some patients receive a single fraction of radiation, while others receive multiple fractions, and it is not clear how these protocols influence the development of anti-tumor immunity. Recently, single fraction RT (compared to surgery alone) for renal clear cell carcinoma patients demonstrated increased tumor associated antigen expression with a concomitant rise in the intratumoral frequency of proliferating CD8^+^ T cells^62^. These parameters will be important to examine in terms of the impact of adrenergic stress.

While our work here reveals that adrenergic stress is a major factor dictating the overall response to radiation in vivo, we have also observed that increased β-AR signaling through treatment with the agonist, isoproterenol, can directly increase tumor cell resistance to radiation in vitro^37^. Thus, how stress affects both direct (tumor cell intrinsic) as well as indirect targets (including anti-tumor immunity as demonstrated here), to regulate the overall radiation response in vivo remains to be established.

Recent studies have demonstrated that an abscopal effect can be induced in patients by the combination of radiotherapy and anti-PD1-therapy (nivolumab^63,64^, pembrolizumab^65,66^), anti-CTLA-4 therapy (Ipilimumab^67^) and granulocyte-macrophage colony-stimulating factor (GM-CSF) therapy^68^. We previously found^29^ that combining Prop with anti-PD-1 in murine tumor models results in a significant improvement in anti-PD-1 efficacy. Thus, here we tested the triple combination of Prop with radiation and anti-PD-1. As we predicted, we found a significantly improved control of tumor growth by radiation and anti-PD-1 when Prop was included. Thus, future work must delineate precise interactions between radiation, immunotherapy delivery, and adrenergic stress in order to maximize therapeutic application of these combination therapies as soon as possible.

In summary, the immunosuppressive impact of cool housing temperature has masked the baseline ability of radiation alone to control tumor growth, especially tumors outside the field of radiation, in essentially all pre-clinical mouse models. However, the study of this phenomenon has led us to recognize the profound role of nerves, stress, and AR signaling in regulating the impact of radiation and provides a useful model with which to explore the impact of the immune system and chronic stress on the efficacy of RT. The data presented here also support the possibility that patients experiencing increased adrenergic stress (elicited by many stressors including anxiety, fear, and depression) following a cancer diagnosis may also be at risk for impaired responses to RT. Related to this possibility, previous retrospective, epidemiological studies show that co-incident use of β-blockers (primarily for cardiovascular conditions) was associated with decreased distant metastases and improved distant metastasis-free survival, disease-free survival, and overall survival in patients with non-small-cell lung cancer after radiotherapy^69,70^, while a recent retrospective study^58^ demonstrates that prior use of non-specific β-blockers enhances survival of patients receiving immunotherapy. These positive results could be linked to enhancement of immunological pathways by stress reduction shown here and strongly support implementation of prospective clinical trials combining β-AR blockade with radiation and/or immunotherapy. Prop is an inexpensive, widely used, and well tolerated drug. Consequently, there are few barriers to implementing many of the studies proposed above in the radiation oncology clinic.

## Methods

### Mice

6–8 weeks old Female BALB/cand C57BL/6 were purchased from Charles River and SCID mice from the Laboratory Animal Resource at Roswell Park Comprehensive Cancer Center. BALB/c mice globally deficient in β2-ARs (*Adrb2*^−/−^) were provided by David Farrar (University of Texas Southwestern Medical Center). Mice were maintained in specific pathogen-free facilities and all studies were performed in accordance with all relevant ethical regulations for animal testing and research as well as the protocols approved by the IACUC at Roswell Park Comprehensive Cancer Center.

### Cell culture and tumor implantation

CT26.CL25 tumor cells (CRL-2639™), B16-F10 tumor cells (CRL-6475™), and 4T1 tumor cells (CRL-2539™) were purchased from and authenticated by American Type Culture Collection (ATCC). 4T1-luc tumor cells were stably tagged with the luciferase gene by Genomics Shared Resource in Roswell Park Comprehensive Cancer Center. CT26.CL25 cells were cultured with Roswell Park Memorial Institute (RPMI) 1640 medium with 2 mM l-glutamine adjusted to contain 1.5 g/L sodium bicarbonate, 4.5 g/L glucose, 10 mM HEPES, 1.0 mM sodium pyruvate, 0.1 mM non-essential amino-acids, 0.4 mg/mL G418, and 10% fetal bovine serum. B16-F10, 4T1-luc, and 4T1 cells were cultured in RPMI 1640 (Gibco) supplemented with 10% FBS, 1% l-glutamine, and 1% penicillin/streptomycin. Once thawed, cells were cultured in an incubator at 37 °C with 5% carbon dioxide and 95% air and passed twice prior to tumor implantation. 5 × 10^5^ CT26.CL25 cells or 1 × 10^5^ B16-F10 cells in 70 μL PBS were subcutaneously injected into both hindlimbs of the mice. For rechallenge experiments, 5 × 10^5^ CT26.CL25 cells or 1 × 10^4^ 4T1 cells were subcutaneously injected into both the hindlimb (in 70 μL PBS, local rechallenge) and in the flank (in 100 μL PBS, distant rechallenge) of the mice cured by the treatment of Prop and radiation for at least 2 months. For lung metastases experiment, 1 × 10^5^ 4T1-luc cells were subcutaneously injected into bilateral 4th mammary fat pad. Tumor growth was monitored by the measurement of perpendicular diameters (width/length) every 2 days and tumor volume was calculated with the formula ((width^2^ × length)/2).

### Irradiation of tumors

When tumors became palpable (~100 mm^3^), one of the tumors per mouse received 6 Gy (CT26), 20 Gy (B16), or 12 Gy (4T1-luc) of 200 kVp x-ray local RT, using a Philips RT-250. The mice were placed in a lead shield to expose one of the tumors and prevent radiation from reaching any other part of the mouse.

### Housing mice at different ambient temperatures

Mice were acclimated to either standard temperature (~22 °C) or thermoneutral temperature (~30 °C) for at least 3 weeks before tumor implantation in precision refrigerated plant-growth incubators (Thermo Fisher Scientific), in which humidity was maintained with a Top Fin® Air Pump AIR 1000 and Top Fin® tubing.

### Treatment of Prop

Mice were intraperitoneally injected daily with 200 μg Prop (P0884, Sigma-Aldrich) in 100 μL of PBS while control mice received 100 μL PBS daily from 3 days prior to irradiation until the endpoint of the experiments.

### Treatment of FTY720

Mice were treated by intragastric administration with 25 μg FTY720 (BML-SL233-0005, Enzo) in 200 μL of PBS while control mice received 200 μL PBS 2 days prior to irradiation and then treated daily with 5 μg FTY720 in 100 μL of PBS while control mice received 100 μL PBS daily until the endpoint of the experiments.

### CD8^+^ and CD4^+^ T cell depletion

Mice were treated weekly with 400 µg *InVivo*MAb anti-mouse CD8α (53-6.72, BioXCell) or *InVivo*MAb anti-mouse CD4 (GK1.5, BioXCell) as experimental groups or with *InVivo*MAb rat IgG2a isotype control (2A3, BioXCell) or *InVivo*MAb rat IgG2b isotype control (LT F-2, BioXCell) as control groups by i.p. injections 4 days prior to tumor implantation. CD8^+^ or CD4^+^ T cell depletion was confirmed by flow cytometry.

### Combination immunotherapy experiment

5 × 10^5^ CT26.CL25 tumor cells were implanted as above-mentioned. Mice were intraperitoneally injected daily with 200 μg Prop (P0884, Sigma-Aldrich) in 100 μL of PBS while control mice received 100 μL PBS daily from 3 days prior to irradiation until the endpoint of the experiments. Six doses of 200 μg anti-mouse PD-1 antibody (RMP1-14, BioXCell) or isotype (2A3, BioXCell) were administered i.p. in 100 μL PBS on days 1, 3, 5, 7, 10, 13 after irradiation.

### Flow cytometry

Mouse tumors were excised and cut into 2–3 mm pieces to create single-cell suspensions. CT26.CL25 and B16-F10 tumors were dissociated with collagenase/hyaluronidase (STEMCELL Technologies, 07912) and murine tumor dissociation kit (Miltenyi, 130-096-730), respectively, following the manufacturers’ recommendation. Dissected LNs were mechanically disrupted to create single cells and washed once using flow running buffer (0.1% bovine serum albumin in PBS). All segregated cells were filtered using a 70 μm nylon cell strainer (Corning).

Resulted single cells were incubated on ice with anti-CD16/32 antibodies for 15 min. The cells were stained using Live/Dead Fixable Violet or Aqua dyes (Thermo Fisher) and surface-labeled with anti-CD8α BUV395 (clone 53-6.7; BD), anti-CD4 BV786 (clone GK1.5; BD), anti-CD3 BV786 or BV605 (clone 145-2C11; BD), anti-CD45 FITC or BV605 (clone 30-F11; BD), anti-PD-1 BV605 (clone J43; BD), anti-CTLA-4 PerCP/Cy5.5 (clone UC10-4B9; BioLegend), anti-Tim3 PE (clone B8.2C12; BioLegend), anti-LAG3 APC (clone C9B7W; BD), anti-CXCR3 BV650 (clone CXCR3-173; BioLegend), anti-CD11b BUV395 (clone M1/70, BD), anti-F4/80 Ax647 (clone T45-2342; BD), and anti-Ly6G BV711 (clone 1A8; BD) for 30 min at room temperature (see Supplementary Table 1 and Supplementary Fig. 8).

For intracellular staining, cells were stimulated with cell activation cocktail containing phorbol myristate acetate (PMA)/ionomycin and Brefeldin A at 37 °C for 4 h prior to surface-staining as described above. The cells were fixed and permeabilized using the forkhead box P3 (FoxP3)/Transcription Factor Staining Buffer Set (eBiosciences) according to the manufacturer’s recommendation. Cells were stained with anti-FoxP3 PE (clone MF23; BD), anti-T-bet Ax647 (clone 4B10; BD), anti-IFN-γ APC (clone XMG1.2; eBioscience), anti-TNFα PerCP/Cy5.5 (clone MP6-XT22; BioLegend), or anti-granzyme B Ax647 (clone GB11; BioLegend). Detailed information pertaining to antibodies and panel used can be found in Supplementary Table 1 and Supplementary Fig. 8.

All events were acquired immediately following sample processing using an LSR Fortessa flow cytometer (BD). All flow cytometric data analysis was performed using FCS Express 6 software package (De Novo Software).

### Multiplex

To determine cytokine protein levels, serum samples from the experimental animals were analyzed using Millipore MCYTOMAG-70K Panel according to manufacturer’s instructions. The plates were run on a Luminex 200 machine and the data analyzed using Upstate BeadView software.

### NanoString

In brief, RNA was isolated from sorted CD8^+^ T cells of irradiated tumors and non-irradiated tumors in CT26 tumor-bearing WT or β2-AR KO mice treated with radiation, using the RNeasy Plus Mini kit (QIAGEN). NanoString analysis was performed with the nCounter Analysis System at NanoString Technologies. The nCounter Mouse Immunology kit was used including 561 immunology-related mouse genes. All data are presented as Log_2_ (fold change of WT/KO) with GraphPad Prism.

### In vivo BLI

On Day 33 post tumor implantation, after surgical removal of the primary tumors, mice were administered 200 μL d-luciferin (150 mg/kg; Caliper Life Sciences) by i.p. and then anaesthetized with isoflurane inhalation prior to imaging. Bioluminescent images of luciferase activity were acquired using BLI system (IVIS Spectrum, PerkinElmer) and analyzed with Living Image 4.3.1 software.

### Histologic analysis of lung metastasis

The lung tissues were fixed in 10% formalin and embedded in paraffin and then stained with hematoxylin/eosin. The number of metastatic lesion was counted by light microscopy.

### Statistical analysis

Student's *t*-test was used to compare data between two groups and tumor growth statistics were calculated using two-way ANOVA with Tukey analysis using GraphPad Prism. One-way ANOVA with Tukey posthoc tests was used to compare data between multiple groups. Differences were considered as statistical significance at *P* < 0.05. All data are presented as mean ± SEM.

## Supplementary information

Supplementary Information

Description of Additional Supplementary Files

Supplementary Data 1

## Data Availability

All relevant data are presented in the article and Supplementary Information. The source data underlying Figs. 1–5 and Supplementary Figs. 1, 3–7 are provided as a Source Data file. All Nanostring data that support the findings of this study have been deposited in the National Center for Biotechnology Information Gene Expression Omnibus (GEO) and are accessible through the GEO Series accession number GSE145262.

## Source Data

Source Data
