# Supplementary material for: Adrenergic stress constrains the development of anti-tumor immunity and abscopal responses following local radiation
Source: Nat Commun. 2020 Apr 14;11:1821. doi: 10.1038/s41467-020-15676-0 (PMC7156731; doi:10.1038/s41467-020-15676-0)
Supplement: Supplementary file 1 — Supplementary Information [file 41467_2020_15676_MOESM1_ESM.pdf]

Supplementary Information

Adrenergic stress constrains the development of anti-tumor immunity and abscopal responses following local radiation

Chen et al.

Corresponding author: Elizabeth A. Repasky, PhD, Department of Immunology, Roswell Park Comprehensive Cancer Center, Buffalo, NY 14263. E-mail: elizabeth.repasky@roswellpark.org.

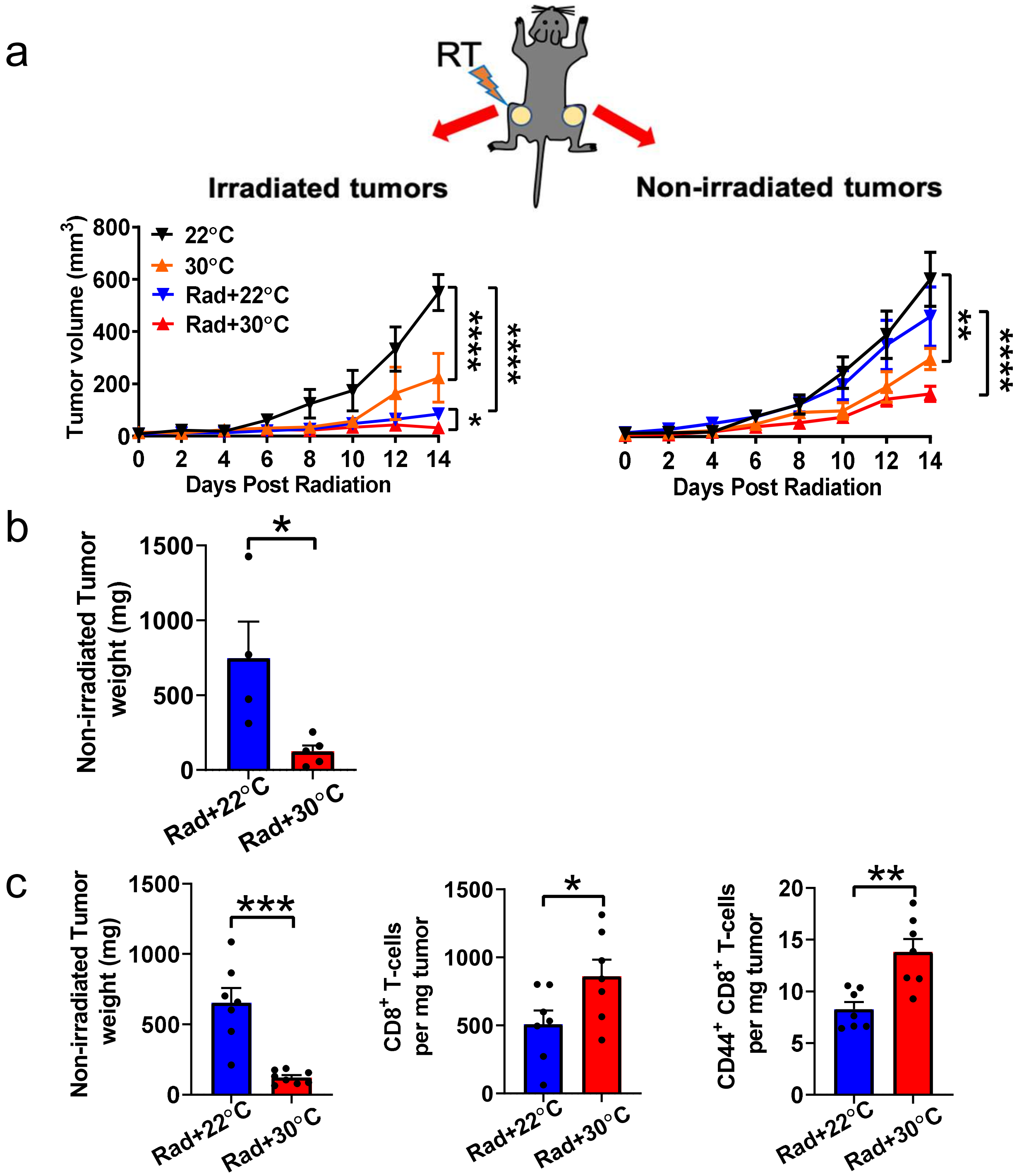

Supplementary Figure 1. Housing mice at a thermoneutral temperature results in an abscopal effect against a tumor contralateral to an irradiated tumor. **a-b** The B16 tumor model. Growth of irradiated tumors (left) and non-irradiated tumors (right) (**a**) and the weight of non-irradiated tumors (**b**) were measured in C57BL/6 mice housed at 22°C or 30°C treated with or without radiation. Data are presented as mean ± SEM. *, *P*<0.05; **, *P*<0.01; ****, *P*<0.0001 (two-way ANOVA analysis for **a**; Student's *t* test analysis for **b**). *n*=4-7 biologically independent mice. (**c**) BALB/c mice were implanted bilaterally with CT26 tumors and housed at 22°C or 30°C. One tumor was irradiated and the non-irradiated tumor was assessed for weight (left), numbers of intratumoral $CD8^+$ T cells (middle), and number of $CD44^+$ $CD8^+$ T cells (right). Data are presented as mean ± SEM. *, *P*<0.05; **, *P*<0.01; ***, *P*<0.001 (Student's *t* test analysis). For **a**, *n*=5 biologically independent mice in 22°C group, *n*=4 biologically independent mice in 30°C group, *n*=7 biologically independent mice in Rad+22°C and Rad+30°C groups; for **b**, *n*=4 biologically independent mice in Rad+22°C group, *n*=5 biologically independent mice in Rad+30°C group; for **c**, *n*=7 biologically independent mice in Rad+22°C and Rad+30°C groups except for *n*=8 biologically independent mice in Rad+30°C group in left panel .

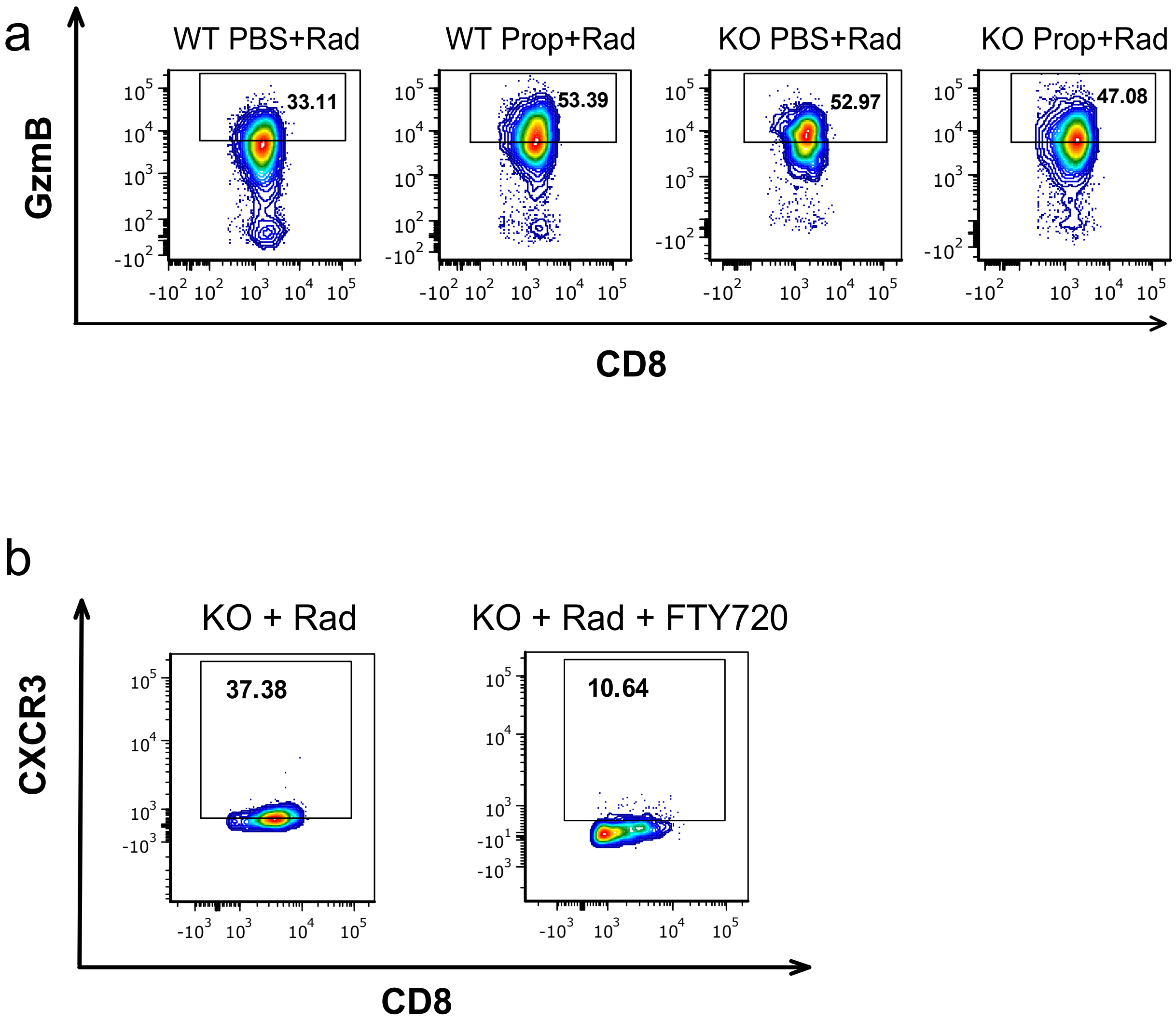

Supplementary Figure 2. Representative flow plots for the data from the CT26 model**.** Tumors were implanted bilaterally and one tumor was irradiated. (**a**) Representative flow plots of GzmB expression in $CD8^+$ T cells in non-irradiated tumors from WT or β2-AR KO mice which were or were not treated with propranolol. (**b**) Representative flow plots of CXCR3 expression in $CD8^+$ T cells in non-irradiated tumors of β2-AR KO treated with or without FTY720.

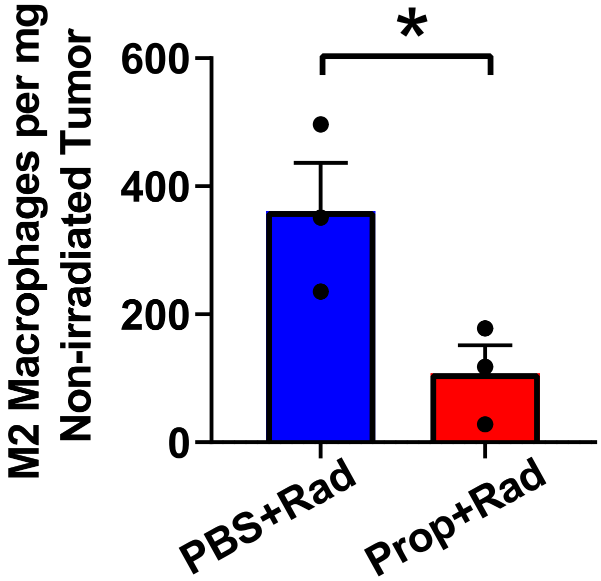

Supplementary Figure 3. β-blocker decreases M2 macrophages ($CD45^{+}CD11b^{+}F4/80^{hi}Ly6G^{-}$) in non-irradiated CT26.CL25 tumors of mice treated with propranolol and radiation compared to the control group. Data are presented as mean ± SEM. *, $P<0.05$ (Student's *t* test analysis). $n=3$ biologically independent mice in two groups.

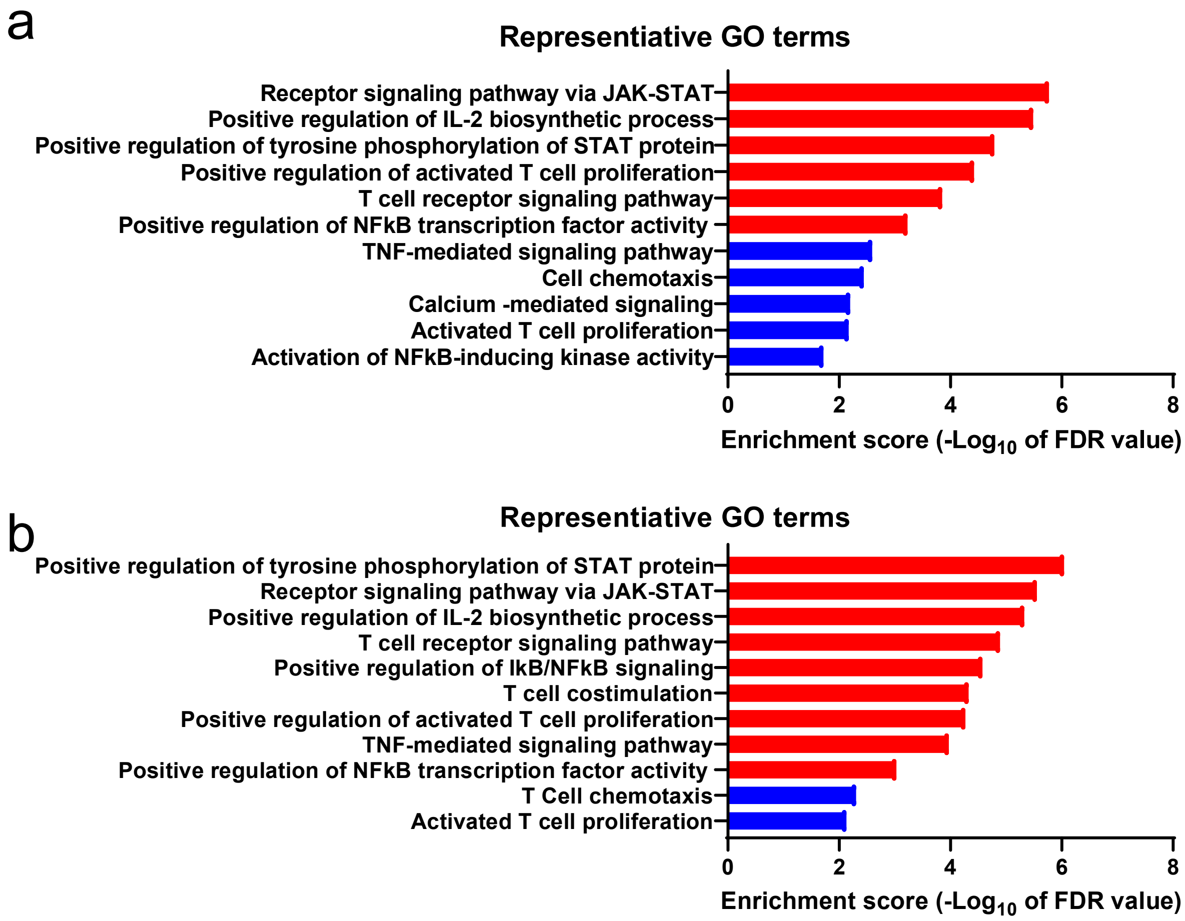

Supplementary Figure 4. Functional annotation of immune-related genes in irradiated β2-AR KO mice. The GO terms (FDR<0.05) of upregulated genes of non-irradiated tumors (**a**) and irradiated tumors (**b**) from irradiated β2-AR KO mice represented as horizontal bars are listed on the y axis and values of $-Log_{10}$(FDR) are shown on the x axis. The red bars represent FDR<10^{-4}. The blue bars represent FDR<0.05.

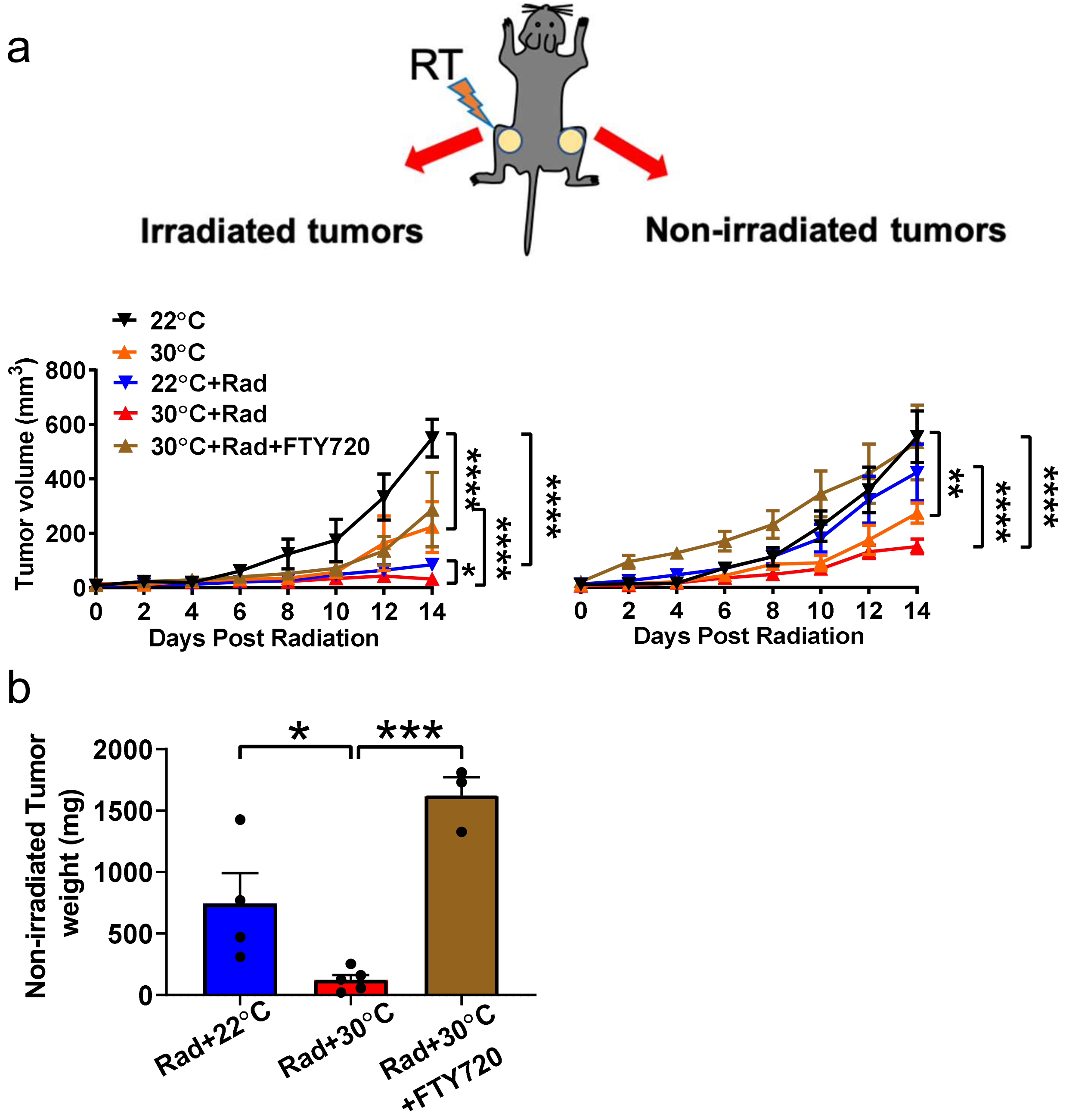

Supplementary Figure 5. Prevention of T-cell migration negates thermoneutral temperature improved abscopal effect of local radiotherapy in B16 melanoma model. The B16 tumor growth of irradiated tumors (left) and non-irradiated tumors (right) (**a**) and the weight of non-irradiated tumors (**b**) were measured in irradiated C57BL/6 mice housed at 22°C or 30°C treated with or without FTY720. Data are presented as mean ± SEM. *, $P<0.05$; **, $P<0.01$; ***, $P<0.001$; ****, $P<0.0001$ (two-way ANOVA analysis for **a**; one-way ANOVA analysis for **b**). For **a**, $n=5$ biologically independent mice in 22°C group, $n=4$ biologically independent mice in 30°C group, $n=7$ biologically independent mice in Rad+22°C and Rad+30°C groups; for **b**, $n=4$ biologically independent mice in Rad+22°C group, $n=5$ biologically independent mice in Rad+30°C group, $n=3$ biologically independent mice in Rad+30°C+FTY720 group.

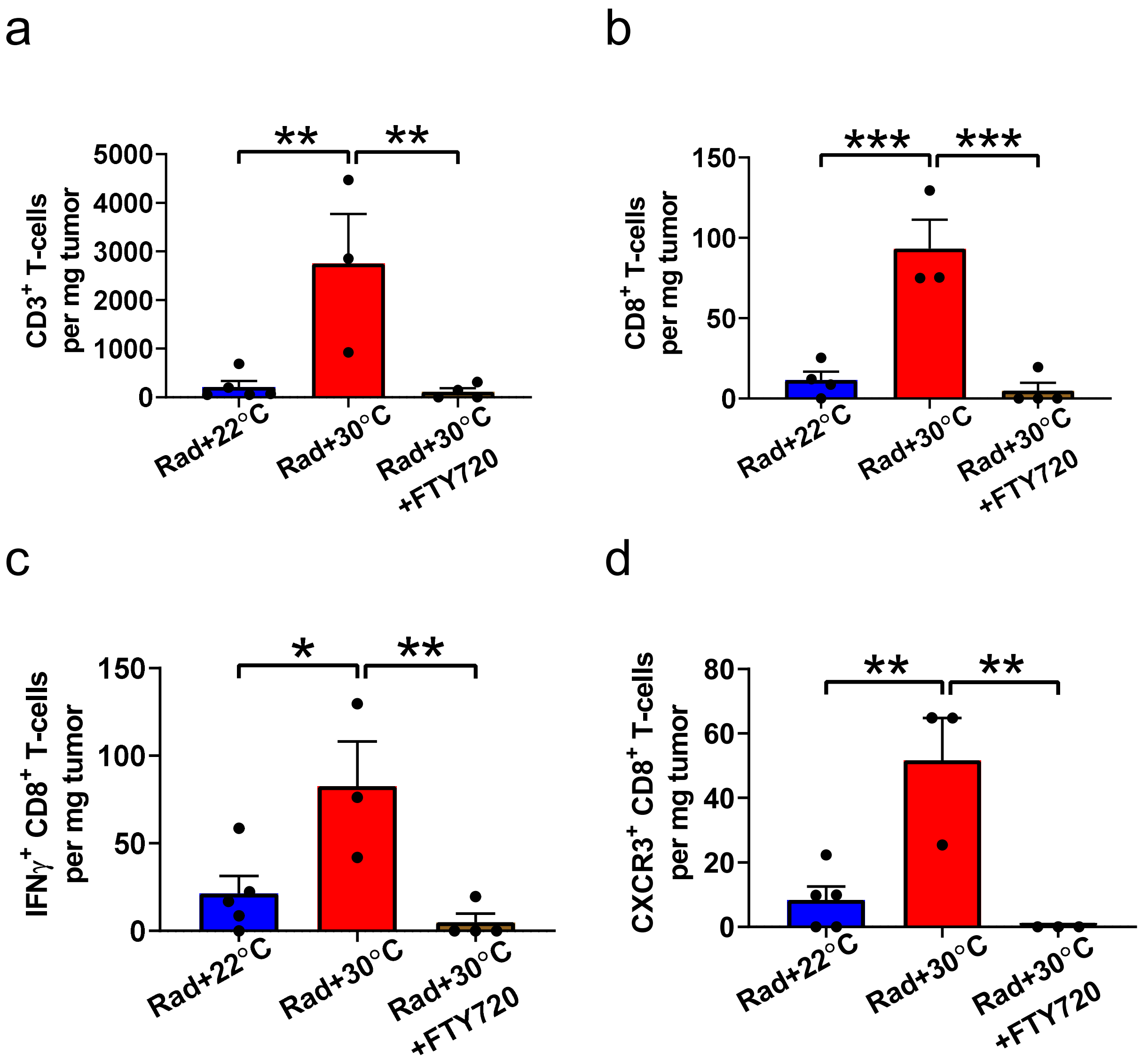

Supplementary Figure 6. Thermoneutral temperature improved abscopal effect of local radiotherapy via enhancing anti-tumor immune response and T-cell egress in B16 melanoma model. CD3$^+$ T cells (**a**), CD8$^+$ T cells (**b**), IFNγ^+ CD8$^+$ T cells (**c**) and CXCR3$^+$ CD8$^+$ T cells (**d**) were detected in non-irradiated tumors (B16) from irradiated mice housed at 22°C or 30°C treated with or without FTY720. Data are presented as mean ± SEM. *, $P<0.05$; **, $P<0.01$; ***, $P<0.001$ (two-way ANOVA analysis). For **a**, n=5 biologically independent mice in Rad+22°C group, n=3 biologically independent mice in Rad+30°C group, n=4 biologically independent mice in Rad+30°C+FTY720 group; for **b**, n=4 biologically independent mice in Rad+22°C group, n=3 biologically independent mice in Rad+30°C group, n=4 biologically independent mice in Rad+30°C+FTY720 group; for **c**, n=5 biologically independent mice in Rad+22°C group, n=3 biologically independent mice in Rad+30°C group, n=4 biologically independent mice in Rad+30°C+FTY720 group; for **d**, n=5 biologically independent mice in Rad+22°C group, n=3 biologically independent mice in Rad+30°C group, n=3 biologically independent mice in Rad+30°C+FTY720 group.

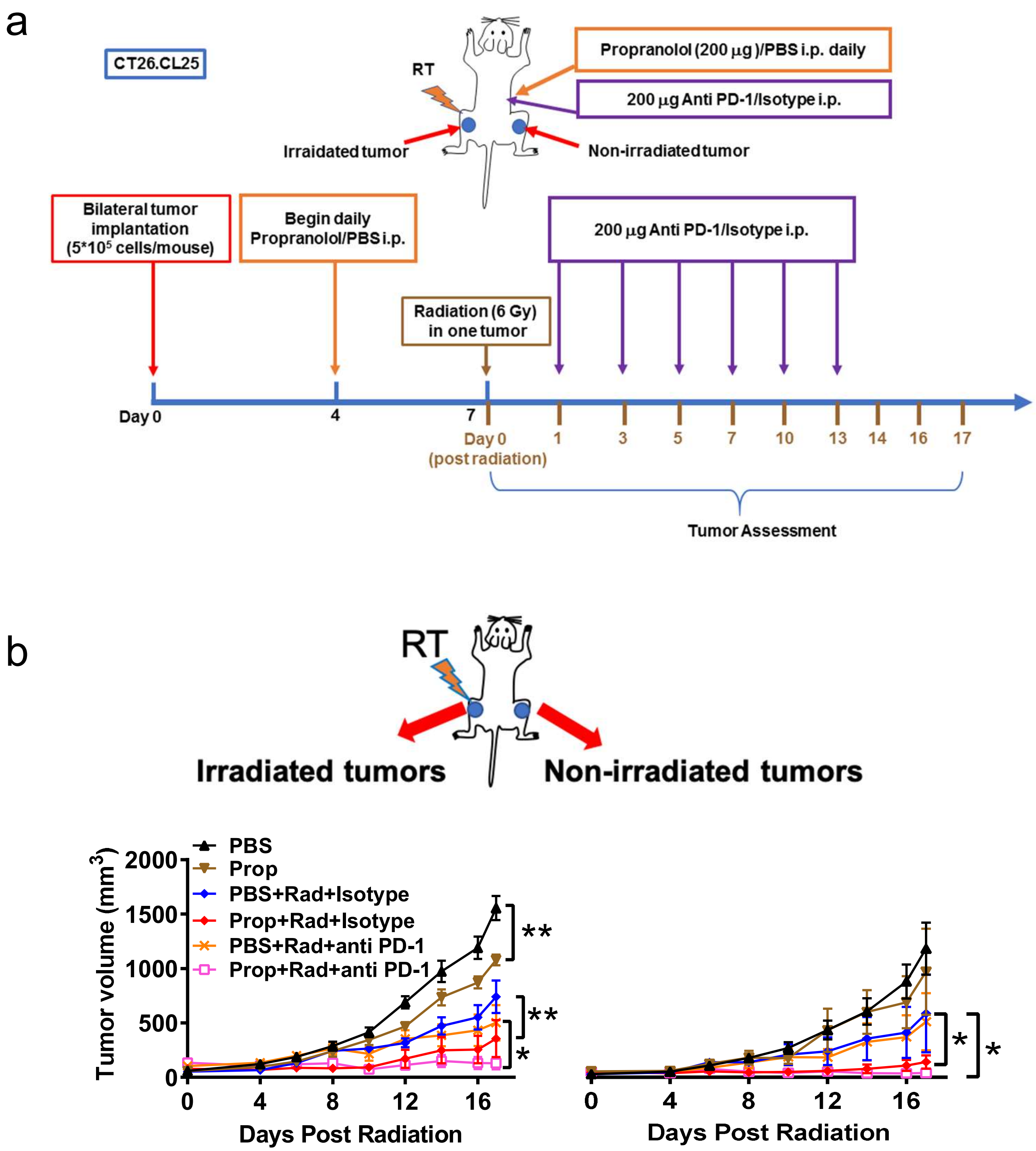

Supplementary Figure 7. β-blocker increases the efficacy of radiation combined with anti PD-1 in CT26 model. (**a**) Experimental design for combination of radiation, propranolol and anti PD-1. Mice housed at 22°C were implanted with CT26.CL25 tumor cells and then randomly received 6 doses of 200 µg anti PD-1 or isotype antibody (Day 1, 3, 5, 7, 10, 13) with or without daily 200 µg propranolol/ PBS treatment. (**b**) Tumor growth is shown for irradiated tumors (left) and non-irradiated tumors (right). Data are presented as mean ± SEM. *, *P*<0.05; **, *P*<0.01 (two-way ANOVA analysis). *n* =7 biologically independent mice in PBS group; *n* =4 biologically independent mice in Prop group; *n* =6 biologically independent mice in PBS+Rad+isotype group; *n* =9 biologically independent mice in Prop+Rad+isotype group; *n* =5 biologically independent mice in PBS+Rad+anti PD-1 group; *n* =6 biologically independent mice in Prop+Rad+anti PD-1 group.

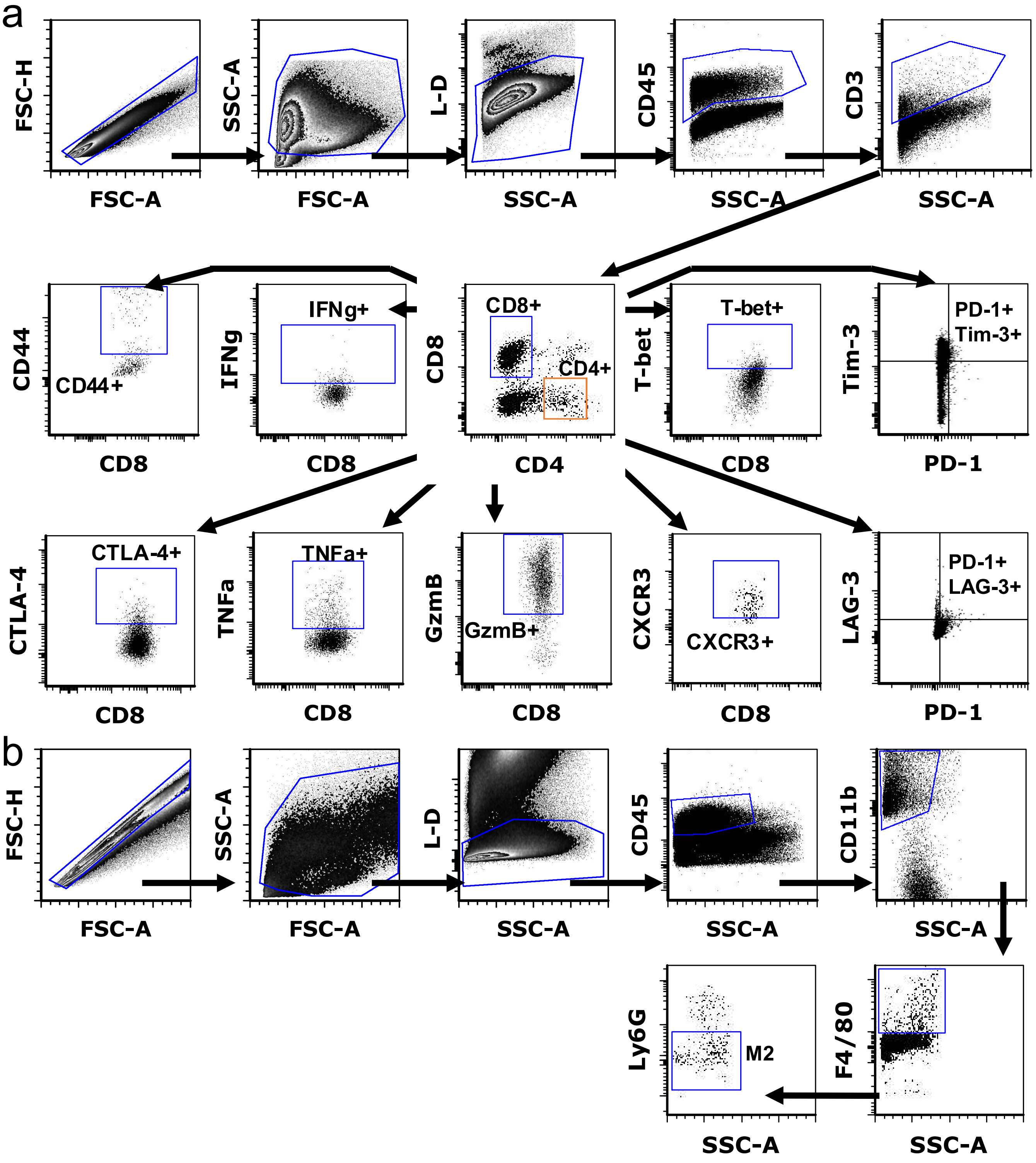

Supplementary Figure 8. Immune profiling of T lymphocytes and M2 macrophages using multiparametric flow cytometry. (**a**) Bivariate plots representing sequential gating approach used for a functional characterization of $CD8^+$ and $CD4^+$ T lymphocytes in non-irradiated tumors and lymph nodes from the mice with specific treatment mentioned in the Methods. Leukocytes that were singlets, debris-free, viable, and $CD45^+$ were identified. Within the leukocyte population, cytotoxic T cells were defined as $CD3^+CD8^+/CD4^-$(Fig. 2a, b, and d; Fig. 3i and j; Fig. 4c; Supplementary Figure 1 and 6), whereas helper T cells were defined as $CD3^+$ $CD4^+/CD8^-$ (Fig. 2d). The expression profiles of IFNɣ$^+$, T-bet$^+$, GzmB$^+$, TNFα$^+$, CXCR3$^+$ (Fig. 3 a-d, g; Fig. 4 d-f, j; Supplementary Figure 2, 6), CTLA-4$^+$, PD-1$^+$Tim-3$^+$, PD-1$^+$LAG-3$^+$ (Fig. 4 g-i) and CD44$^+$ (Supplementary Figure 1c) were evaluated in CD8$^+$ T cells. (**b**) Identification of M2 macrophages. Myeloid cells that were singlets, debris-free, viable, CD45$^+$, and CD11b$^+$ were identified. Within myeloid cells, M2 macrophages were defined as F4/80, Ly6G$^-$ (Supplementary Figure 3).

Supplementary Table 1 Detailed information on commercial antibodies

Number	Name	Company	Cat.No.	Dilution
1	anti-CD8α BUV395	BD	563786	1:160
2	anti-CD4 BV786	BD	563331	1:100
3	anti-CD3 BV786	BD	564010	1:160
4	anti-CD3 BV605	BD	564009	1:160
5	anti-CD45 FITC	BD	553080	1:100
6	anti-CD45 BV605	BD	563053	1:100
7	anti-CD11b BUV737	BD	564443	1:100
8	anti-F4/80 Ax647	BD	565853	1:100
9	anti- Ly6G BV711	BD	563979	1:80
10	anti-PD-1 BV605	BD	563059	1:50
11	anti-CTLA-4 PerCP/Cy5.5	Biolegend	106316	1:80
12	anti-Tim3 PE	Biolegend	134004	1:80
13	anti-LAG3 APC	BD	562346	1:50
14	anti-CXCR3 BV650	Biolegend	126531	1:50
15	anti-FoxP3 PE	BD	560408	1:80
16	anti-T-bet Ax647	BD	561264	1:20
17	anti-IFN-γ APC	eBiosciences	17-7311-82	1:160
18	anti-TNFa PerCP/Cy5.5	Biolegend	506322	1:80
19	anti-granzyme B Ax647	Biolegend	515406	1:20
20	BV605 Hamster IgG2, k Isotype Control	BD	563012	1:50
21	PerCP/Cy5.5 Armenian Hamster IgG Isotype Ctrl Antibody	Biolegend	400931	1:80
22	PE Rat IgG1, k Isotype Ctrl Antibody	Biolegend	400407	1:80
23	BV650 Armenian Hamster IgG Isotype Ctrl Antibody	Biolegend	400945	1:50
24	PE Rat IgG1 k Isotype Control	BD	551979	1:80
25	Ax647 mouse IgG1 k Isotype Control	BD	557783	1:20
26	APC Rat IgG1 k Isotype Control	eBioscience	17-4301-81	1:160
27	PerCP/Cy5.5 Rat IgG1 k Isotype Ctrl antibody	Biolegend	400425	1:80
28	Ax647 Mouse IgG1 k Isotype Ctrl antibody	Biolegend	400135	1:20
