# Supplementary material for: Adrenergic stress constrains the development of anti-tumor immunity and abscopal responses following local radiation
Source: Nat Commun. 2020 Apr 14;11:1821. doi: 10.1038/s41467-020-15676-0 (PMC7156731; doi:10.1038/s41467-020-15676-0)
Supplement: Supplementary file 2 — Description of Additional Supplementary Files [file 41467_2020_15676_MOESM2_ESM.docx]

**Description of Additional Supplementary Files**

**File Name:** Supplementary data 1

**Description:** Differences in gene expression between CD8+ T-cells from tumors in WT mice vs. β2-AR KO mice.
